# Characterization of the complete plastid genome of Chinese medicinal plant *Isodon serra* (Lamiaceae)

**DOI:** 10.1080/23802359.2020.1765429

**Published:** 2020-05-18

**Authors:** Hui-Ye Zhang, Wen-Zhe Ma, Hai-Fei Yan, De-Qin Wang

**Affiliations:** a Hutchison Whampoa Guangzhou Baiyunshan Chinese Medicine Co., Ltd, Guangzhou, China; b Guangdong AIB Polytechnic College, Guangzhou, China; c Key Laboratory of Plant Resources Conservation and Sustainable Utilization, South China Botanical Garden, The Chinese Academy of Sciences, Guangzhou, China

**Keywords:** Lamiaceae, isodon, plastome, phylogeny

## Abstract

The herb *Isodon serra* (Maximowicz) Kudô, which is widely distributed in China and its neighbor regions, is a well-known traditional Chinese medicinal plant. In this study, we characterized the complete plastid genome sequence of *I. serra* using Illumina sequencing data. The plastome is 152,676 bp in length and contains a typical quadripartite structure. The inverted repeat (IR), large-single copy (LSC) and small-single copy (SSC) regions each has 25,716 bp, 83,564 bp, and 17,680 bp. The genome contains 80 protein coding genes (PCGs), 30 transfer RNAs (tRNA), and four ribosomal RNAs (rRNA). The phylogenetic result indicates *I. serra* together with genera *Ocimum* and *Lavandula* formed tribe Ocimeae clade.

The genus *Isodon* (Schrad. ex Benth.) Spach (Lamiaceae) comprises ca. 100 species, and is widely distributed from tropical and subtropical Asia to southern Africa (Li and Hedge 1994; Yu et al. 2014). Many *Isodon* species are used in popular folk medicine in China, such as *Isodon rubescens* (Sun et al. 2006). The herb *Isodon serra* (Maximowicz) Kudô, widely distributed in China and its neighbor regions (Li and Hedge 1994), is a well-known traditional Chinese medicinal plant for its antibacterial, anti-inflammatory and antitumor activities (Huang et al. 2019). To our knowledge, the *Isodon* plastid genome has not been reported in the literature. Herein, we characterized the complete plastid genome sequence of *I. serra* using Illumina sequencing data. The plastid genome (GenBank accession no. MT317099) will be useful in the evolutionary and medicinal studies of *Isodon.*

Fresh leaves of an individual of *I. serra* was collected from Hutchison Whampoa Guangzhou Baiyunshan Chinese Medicine Co., Ltd (N23°11′11″, E113°15′57″), and used to extracted Genomic DNA using a modified CTAB protocol (Doyle and Doyle 1987). The voucher specimen is deposited at the Herbarium of South China Botanical Garden (Herbarium code: IBSC) with the accession number Zhy-Z3. Illumina paired-end library was constructed and sequencing based on Illumina Hiseq X Ten platform at Beijing Genomics Institute (Wuhan, China). The genomes were assembled using SPAdes v3.10.1 (Bankevich et al. 2012) and Geneious Prime 2019 (Biomatters, Ltd, Auckland, New Zealand) was subsequently used to close gaps. The coding and tRNA genes were annotated using Geneious Prime 2019. The maximum likelihood (ML) tree was reconstructed using RAxML (Stamatakis 2014) with the GTR + gamma model and 1000 bootstraps.

The plastome of *I. serra* is 152,676 bp in length and contains a typical quadripartite structure, comprising two copies of inverted repeat (IR) regions (25,716 bp), a large-single copy (LSC) region (83,564 bp), and a small-single copy (SSC) region (17,680 bp). It has 80 protein coding genes (PCGs), 30 transfer RNAs (tRNA), and four ribosomal RNAs (rRNA). The GC content of the genome is 37.6%, and varies among three regions. Specifically, LSC, SSC, and IR regions each has 35.7, 31.1, and 43.1%. The ML tree based on 80 PCGs shows that tribe Elsholtzieae (only represented by *Perila* in this study) is sister to the remainder of the subfamily Nepetoideae, followed by a sister group tribe Ocimeae + tribe Mentheae. *I. serra* together with genera *Ocimum* and *Lavandula* formed tribe Ocimeae clade (Figure 1). This evolutionary relationships recovered here are consistent with the most recent analyses (Chen et al. 2016; Li et al. 2016).

**Figure 1.**
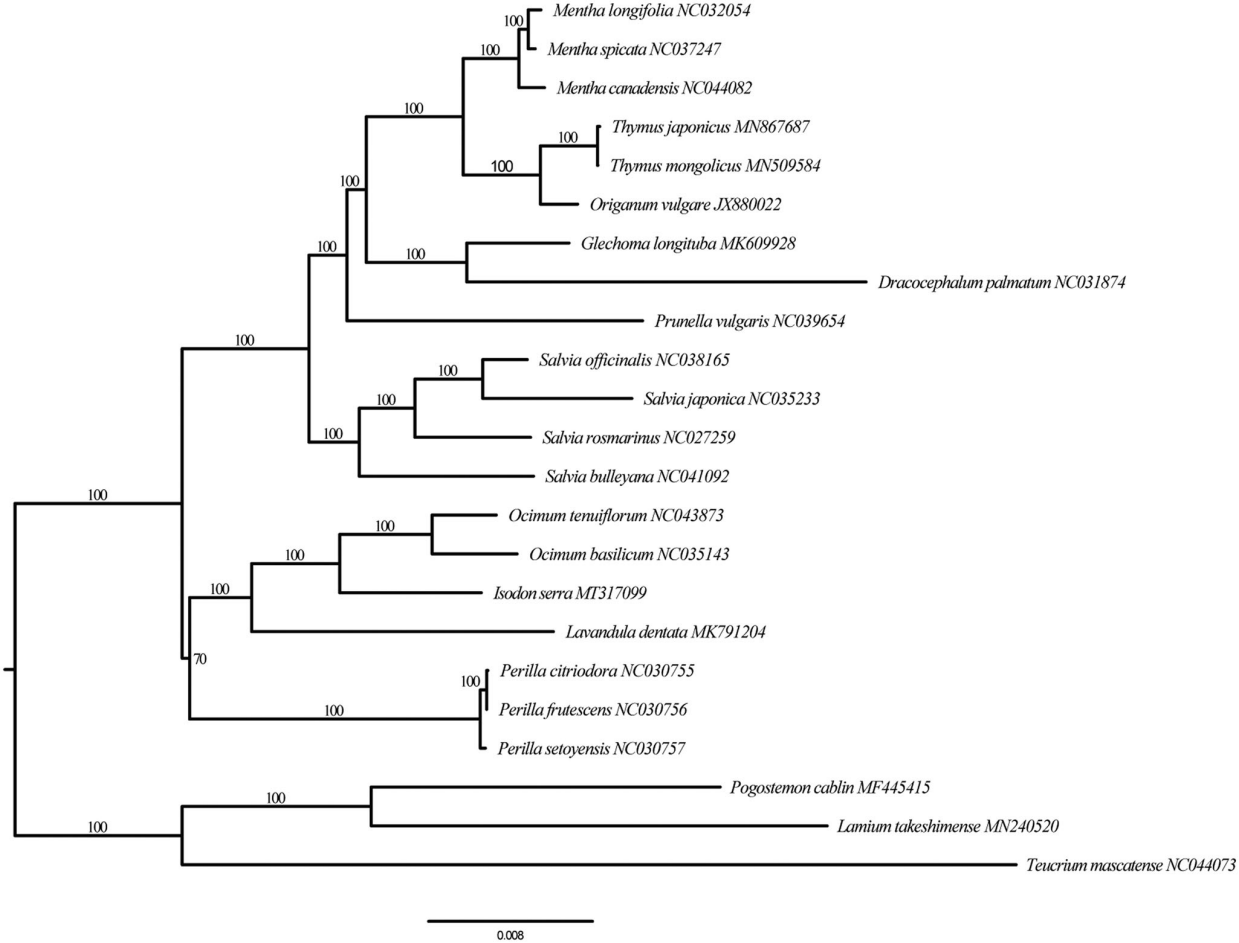
Maximum likelihood tree of *Isodon serra* and related species in the subfamily Nepetoideae based on 80 PCGs. Numbers along branches are RAxML bootstrap supports based on 1000 replicates. The scale for nucleotide substitutions is showed in legend below.

## Data Availability

The data that support the findings of this study are openly available in GenBank at https://www.ncbi.nlm.nih.gov/, reference number MT317099.
